# How to build better memory training games

**DOI:** 10.3389/fnsys.2014.00243

**Published:** 2015-01-09

**Authors:** Jenni Deveau, Susanne M. Jaeggi, Victor Zordan, Calvin Phung, Aaron R. Seitz

**Affiliations:** ^1^Department of Psychology, University of California, RiversideRiverside, CA, USA; ^2^School of Education, University of California, IrvineIrvine, CA, USA; ^3^Department of Cognitive Sciences, University of California, IrvineIrvine, CA, USA; ^4^Department of Computer Science, University of California, RiversideRiverside, CA, USA

**Keywords:** working memory, video games, brain training, perceptual learning, game design

## Abstract

Can we create engaging training programs that improve working memory (WM) skills? While there are numerous procedures that attempt to do so, there is a great deal of controversy regarding their efficacy. Nonetheless, recent meta-analytic evidence shows consistent improvements across studies on lab-based tasks generalizing beyond the specific training effects (Au et al., 2014; Karbach and Verhaeghen, 2014), however, there is little research into how WM training aids participants in their daily life. Here we propose that incorporating design principles from the fields of Perceptual Learning (PL) and Computer Science might augment the efficacy of WM training, and ultimately lead to greater learning and transfer. In particular, the field of PL has identified numerous mechanisms (including attention, reinforcement, multisensory facilitation and multi-stimulus training) that promote brain plasticity. Also, computer science has made great progress in the scientific approach to game design that can be used to create engaging environments for learning. We suggest that approaches integrating knowledge across these fields may lead to a more effective WM interventions and better reflect real world conditions.

## Introduction

As long as scientists have explored memory, they have strived, and often failed, to improve it. Most approaches to improve memory implement strategies, such as creating mnemonic devices (for example, the method of loci). However, despite evidence these techniques improve memory *performance*, they do not target underlying memory *processes*, and while they do have some influence on memory systems in the brain (Maguire et al., 2003), they typically fail to broadly generalize to untrained activities (Verhaeghen et al., 1992; Maguire et al., 2003; St Clair-Thompson et al., 2010). Recent research on “brain training” renews promise for improving memory and other cognitive skills. Here, we focus on working memory (WM), a limited-capacity system for storing and manipulating information in a given moment. WM underlies performance in virtually all complex cognitive tasks (Shah and Miyake, 1999). Recent approaches targeting skills related to WM (Klingberg et al., 2002, 2005; Jaeggi et al., 2008, 2010; Anguera et al., 2013; Goldin et al., 2014) have shown generalizing benefits to a wide number of non-trained cognitive tasks that are thought to rely on WM, including executive control and fluid reasoning (c.f. Au et al., 2014; Karbach and Verhaeghen, 2014 for recent meta-analyses).

Here we review recent WM training approaches discussing their strengths and limitations and suggest methods that are based on the principles of perceptual learning (PL) and game design to make them more effective. Off-the-shelf computer games and standard cognitive approaches each contain component properties that can benefit WM. We propose that integrating knowledge from psychology and neuroscience along with the science of video game design could critically inform the development of engaging, cognitively immersive challenges that will more optimally train WM memory processes.

## Span training

Span training targets WM capacity (Klingberg et al., 2002, 2005) typically relying on two types of tasks, simple and complex. Simple span tasks present sequences of stimuli that vary in set-size with participants typically reporting the items in (reverse) order of presentation. Research has shown that training on simple span tasks results in transfer in a variety of measures, such as non-trained WM tasks, response inhibition, and even fluid reasoning (Klingberg et al., 2002, 2005; Thorell et al., 2009).

**Figure 1:**
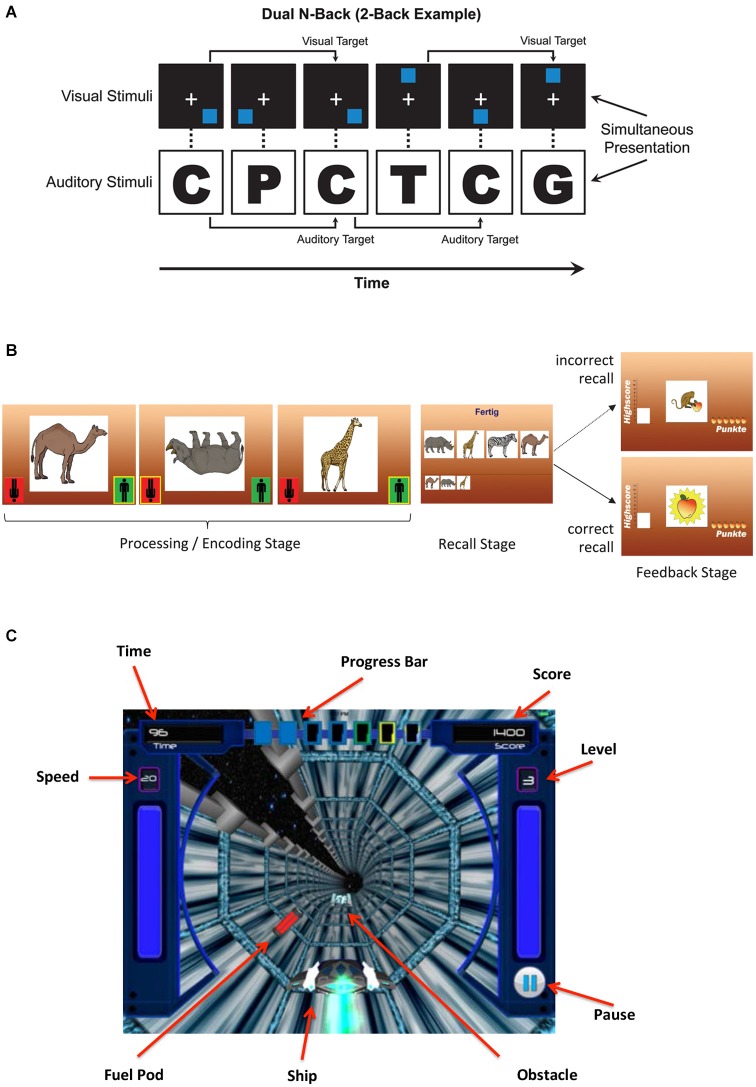
**(A)** Diagram of an n-back task presented as a 2-back task (Jaeggi et al., 2008, 2010). Here, visual and auditory stimuli are presented simultaneously and participants have to respond to both modality streams independently. **(B)** Diagram of a complex span training task (Buschkuehl et al., 2008; Loosli et al., 2012). Animal pictures are presented sequentially, and participants respond to the orientation of each picture, and then reproduce the sequence in which the animals were presented. **(C)** Schematic of gamified n-back training task.

In contrast to simple span tasks, which focus predominantly on WM storage, complex span tasks involve a secondary processing task. An example of a complex span task is Reading Span (Daneman and Carpenter, 1980) where participants judge the semantic content of a series of sentences and then later recall the last word of each sentence in order. Simple and complex span tasks used as interventions are typically adaptive, where the number of items to be recalled increases as training progresses. Adaptive complex span training leads to both near and far transfer in a variety of populations. Chein and Morrison (2010) showed that training on verbal and spatial complex span tasks improves verbal and spatial short-term memory, response inhibition, and reading comprehension in young and older adults (Richmond et al., 2011). In another series of studies, typically developing children (Loosli et al., 2012; Karbach et al., 2014) and older adults (Buschkuehl et al., 2008) were trained on complex span (see Figure 1B) demonstrating improved reading performance (Loosli et al., 2012; Karbach et al., 2014) and improved visual WM and episodic memory (Buschkuehl et al., 2008). Other groups have found similar effects from complex span training in older adults that were maintained several months after training completion (Borella et al., 2010, 2013, 2014).

## N-back training

Among the best known WM tasks used for training is the n-back task (Jaeggi et al., 2008; Smith et al., 2009; Buschkuehl and Jaeggi, 2010). Here, participants memorize and constantly update the serial positions “*n* steps back” in a continuous stimulus stream and report whether or not the current stimulus matches the stimulus *n*-items back in a sequence (Figure 1A). Task difficulty (equal to level of *n*) is adaptively adjusted in response to participants’ performance. N-back training-related improvements are wide-ranging, including non-trained WM functions (e.g., Lilienthal et al., 2013), executive functioning (e.g., Salminen et al., 2012), episodic memory (e.g., Rudebeck et al., 2012), and even fluid reasoning (Stephenson and Halpern, 2013). Effects are observed across the lifespan ranging from typically developing children (Jaeggi et al., 2011) to older adults (Stepankova et al., 2014). Both the amount of training (Stepankova et al., 2014) and achievement on the training task (Jaeggi et al., 2011) have been related to consequent improvements in the untrained fluid intelligence tasks. However, findings of far transfer (e.g., that WM training leads to improved performance on tasks unrelated to the training), are not ubiquitous in the literature (Shipstead et al., 2012). Some of these inconsistencies across studies may be explained by variations in training schedules, outcome measures, or individual differences (Jaeggi et al., 2012, 2014; Shah et al., 2012).

## Mechanisms that promote learning

While there is growing evidence that WM training impacts performance in a wide variety of tasks, the mechanisms driving plasticity in WM systems remain unclear. However, research of PL has identified numerous mechanisms that promote the magnitude and generalization of learning. PL refers to a long lasting improvement in perceptual abilities as a result of experience. Interestingly, key approaches to working WM training, such as extended practice and adaptive procedures (the latter is to use computer algorithms to customize the challenge to each participant), were originally modeled after successful approaches in PL (Klingberg et al., 2002, 2005). Classically, a translational barrier to PL has been its high degree of specificity to trained stimulus features (Fahle, 2005), such as orientation (Fiorentini and Berardi, 1980), retinal location (Karni and Sagi, 1991) or the eye trained (Poggio et al., 1992; Seitz et al., 2009). This specificity to the training task and stimuli mirrors issues that face modern WM training. However, recent research illustrates how to overcome this “curse of specificity” with approaches that integrate many techniques (Deveau et al., 2014a,b) showing greater generalization of learning.

A key question is what mechanisms gate learning? Seitz and Dinse (2007) proposed a model of PL in which mechanisms including attention, reinforcement, optimal stimulation protocols, and multisensory facilitation interact to boost sensory signals over a learning threshold. This model and a host of empirical research on PL demonstrate that learning generalizes best when: (1) a larger set of stimulus features are trained (Xiao et al., 2008; Hung and Seitz, 2014); (2) using multisensory stimuli (Shams and Seitz, 2008); (3) using motivating tasks (Shibata et al., 2009); (4) participants are confident in their performance (Ahissar and Hochstein, 1997); and (5) consistent reinforcement to the training stimuli is used (Seitz and Watanabe, 2009). Combining these approaches increase the magnitude and generality of learning (Deveau et al., 2014a,b). In the following, we review some of the mechanisms that promote PL and discuss how they could be applied to WM training.

## Attention and reinforcement

Attention refers to a set of mental processes that selectively modulate the processing of relevant information over irrelevant information; attention influences decisions, guides memory processes and our executive functions to direct resources to act upon the world. Numerous studies show that attention gates learning (Shiu and Pashler, 1992; Ahissar and Hochstein, 1993; Schoups et al., 2001; Leclercq and Seitz, 2012b). For example, Schoups et al. (2001) found neuronal plasticity of V1 cells corresponding to attended stimuli but no plasticity for cells with receptive fields overlapping unattended stimuli, suggesting that attention selects what is learned and what is not. A key aspect of WM capacity entails the ability to avoid distraction and is positively correlated with performance on a variety of attention tasks (Engle, 2002; Hutchison, 2007). Furthermore, WM capacity is highly predictive for scholastic achievement (Gathercole et al., 2003) and it is among the major cognitive deficits of children with attention deficit hyperactivity disorder (ADHD; Klingberg et al., 2002). Using casual games found on the Internet, Baniqued et al. (2013) found that playing games focusing on attention/object tracking improved WM abilities, on the other hand, playing games that focused on WM did not improve measures of attention. These and other findings suggest a key role of attention in WM, and that proper engagement of attention during training may be a key factor in WM training success.

Also, reinforcement processes (rewards, punishments, motivation, etc.) have fundamental importance in guiding learning. For example, Seitz et al. (2009) found improved discrimination of orientation stimuli masked in noise after temporal-pairing between a liquid reward and a subliminal presentation of that orientation stimulus. Seitz and Watanabe (2005) suggested a model where learning is gated by reinforcement signals that trigger learning of aspects of the environment (even those for which the organism is not consciously aware) that are predictive or co-vary with the reinforcing event. They suggested that both attention and reinforcement operate in part through the release of neuromodulatory signals in the brain. For example, the orienting of attention, in the direction of the target-arrow, has been linked with the acetylcholine neuromodulatory system (Davidson and Marrocco, 2000). Of interest, cholinergic enhancement through the use of donepezil improves both the attentional processing (Rokem et al., 2010) as well as the magnitude (Rokem and Silver, 2010) and longevity (Rokem and Silver, 2013) of PL. Other neuromodulatory systems, such as dopamine and norepinephrine have also been linked to attention (Posner and Petersen, 1990; Fan et al., 2003), learning (Kilgard and Merzenich, 1998; Bao et al., 2001; Dalley et al., 2001; Blake et al., 2006) and WM (Brehmer et al., 2009; Bellander et al., 2011). These studies suggest that a good training approach should involve the direction of both attention and reinforcement in a coordinated manner to promote learning.

## Multisensory facilitation

The human brain has evolved to learn and operate optimally in natural environments in which behavior is guided by information integrated across multiple sensory modalities. Crossmodal interactions are ubiquitous in the nervous system and occur even at early stages of perceptual processing (Shimojo and Shams, 2001; Calvert et al., 2004; Schroeder and Foxe, 2005; Ghazanfar and Schroeder, 2006; Driver and Noesselt, 2008). For example, recent research shows that subjects trained with auditory-visual stimuli exhibit a faster rate of learning and a higher degree of improvement than found in subjects trained in silence (Seitz et al., 2006; Kim et al., 2008).

Both memory storage and retrieval involves multiple senses. For example, the smell of a loved one’s perfume (olfaction) can invoke memories of their face (vision), and so on. However, existing memory tasks, such as the dual *n*-back, often employ multiple (stimulus) modalities that are not coordinated, which research shows does not promote learning, and may in fact interfere with it (Seitz et al., 2005). Instead, we suggest a different approach where multisensory objects are incorporated into WM training. By defining objects through multi-modal feature sets, each sense can boost learning in the other. For example, an individual with limited visual capabilities will benefit from training utilizing concordant auditory stimuli.

## Multistimulus training

One way to overcome specificity of learning is by training with multiple stimuli, as demonstrated in PL (Dosher and Lu, 1998; Yu et al., 2004; Xiao et al., 2008; Deveau et al., 2014a,b). For example, the recently developed technique of “double training” found that classically specific learning effects can show broad transfer when more than one stimulus attribute is trained. Xiao et al. (2008) trained participants on a Vernier discrimination task at a specific orientation at a specific location in the visual field, which normally yields location and orientation specific learning (Poggio et al., 1992). However, subsequently training subjects a second orientation at a different spatial location, the training-induced changes for the second orientation transferred to the first location. This data suggests that WM training with a diverse stimulus set might lead to a greater degree of transfer to untrained tasks than training on a narrow set (Estes and Burke, 1953; Schmidt and Bjork, 1992).

**Figure 2:**
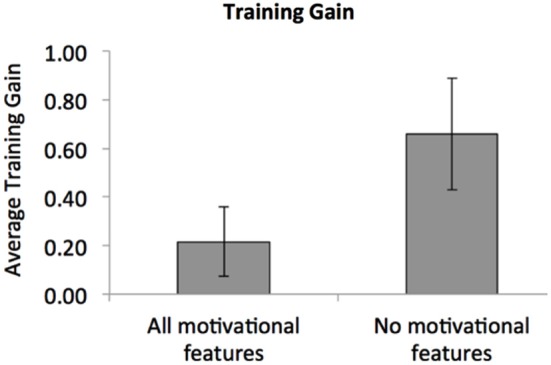
**Interference of learning by gamification**. Motivational features such as scores, prizes, and scene-changes seem to interfere with learning. Specifically, training with all these features led to a lesser degree of learning compared to training without motivational features over the course of three sessions of *n*-back training (adapted from Katz et al., 2014).

Furthermore, PL shows that the arrangement of multiple task elements in space and time can play a pivotal role in determining learning. Poor arrangements, such as when different stimuli are presented in a random order, as opposed to fixed order (Zhang et al., 2008), lead to poor learning (Seitz et al., 2005). Similar rules operate in guiding memory where sudden onsets of task-related stimuli can disrupt memorization of objects paired with those images (Leclercq and Seitz, 2012a). This may explain the recent counter-intuitive finding of WM training where the addition of motivational features in a simple gamification of n-back training led to impaired learning (Katz et al., 2014; see Figure 2), by inadvertently leading to greater distraction. We suggest that greater congruence between training stimuli and motivational factors will lead to greater and broader learning from WM training. In the next section we describe how this may be achieved through game design principles.

## Video games

There are many examples of off-the-shelf video games leading to substantial improvements in a variety of perceptual and cognitive abilities. For example, Green and Bavelier (2003) found that training novices for 10 h on an action video game improved performance on enumeration, useful field of view, and attentional blink tasks when compared to participants trained with a non-action video game. Basak et al. (2008) found that playing a real-time strategy game improved executive control as measured by task switching, visual short-term memory, and reasoning in older adults. Another recent study (Shute et al., 2015) showed that an off-the-shelf video game (Portal 2) led to substantial improvements on measures of problem solving, spatial skills, and persistence (in fact, even more so than training with the popular brain training games of Lumosity). Furthermore, dyslexic children improved reading speed and attentional abilities after playing an action video game (Franceschini et al., 2013). Finally, Goldin et al. (2014) employed several computer games targeting executive control, and found improvements in attention, inhibitory control, and planning, which also translated to school performance (Goldin et al., 2014). Together these studies suggest that video games include important attributes that contribute to learning.

Given the attractive motivational features of video games, recent research in cognitive science is increasingly moving towards adding game-like elements to their assessments. However, without proper design these can impair task performance, and even weaken test quality and learning (Hawkins et al., 2013; Katz et al., 2014). We suggest a better approach is to create training software that will dovetail, and/or implement non-competing concepts from game design that support learning. The video-game field is maturing, proper design rules and constraints are becoming more refined and the practices of coordinated design are becoming better understood and documented (Rabin, 2005). For example, in order to optimally engage players games must establish clear goals and allow players to realize those goals through meaningful actions (Salen and Zimmerman, 2004). Successful game design has critical aspects that make software engaging, including its mechanics, interaction, visual/sensory experience, and progression (Gee, 2007).

Many game design criteria mirror components found to improve learning from the PL literature, and literature on deliberate practice (Ericsson et al., 1993). For example, consistent reinforcement to training stimuli (Seitz and Watanabe, 2009) maps directly to consistent player feedback, a key part of *player-centric interface design* (Adams, 2009). Adams says (of players) “most critically, they need information about whether their efforts are succeeding or failing, taking them closer to victory or closer to defeat”. Likewise, motivating tasks (Shibata et al., 2009) and ensuring subjects are confident of their performance (Ahissar and Hochstein, 1997) are consistent with good game-design principles such as establishing clear goals (Salen and Zimmerman, 2004) and balancing games challenges (related to the adaptive approaches used in PL and WM training) to match player performance (Adams, 2009). Applying video-game techniques purposefully into WM training can inject the cognitive benefits found from off-the-shelf video games into principled cognitive training, while also being fun to play.

## Integrating learning and gaming principles

Two relevant lines of research have made significant breakthroughs in brain training: (1) Studying incidental benefits of off-the-shelf video games; and (2) Transforming standard cognitive tasks into training tasks. We suggest that the most success will come from integrating knowledge of memory systems with that of brain plasticity and modern game-design principles.

As a first attempt to implement this approach, we created a prototype game that incorporates mechanics of the n-back into an engaging 3D space-themed game^1^ (see Figure 1C). Typically, the n-back task is very basic, e.g., selecting matches from a grid or a picture series. In contrast, our prototype is a space-themed “collection” game with navigation challenges and obstacles, multi-layered progression through levels, and rich, thematic visual and sound effects. The n-back task is integrated into the game mechanics, where players select the “right” fuel cells while avoiding decoys. Levels are designed to get progressively harder through increasing cognitive challenge (n-level) and other game challenges (such as obstacles). While the game is more difficult and attention is spread over more elements than the conventional n-back, participant’s control over their environment is anticipated to increase their engagement with the game.

The game also incorporates principles from PL, where participants are trained on multisensory (auditory and visual) features, where sounds and visuals are designed to facilitate each other, and where attention and reinforcement are carefully sculpted to lead to the best learning. While much work is still required to maximize the game’s efficacy, e.g., by incorporating a broader stimulus set, adding other memory tasks, and creating an even more compelling game framework, we put it forward as a first example of how to build such an integrative game. Initial piloting with our prototype indicates participants are engaged in the game and improve performance (n-level) across training sessions. However, more research is needed to make firm conclusions regarding its transfer potential.

In summary, we suggest that more integrative approaches will lead to better learning outcomes. We suggest that the general mechanisms that promote PL are shared across brain regions and will also promote WM. Furthermore, there is enough known about the aspects of conventional video games that lead to positive learning outcomes that these principles can be applied to achieve more effective WM training. Additionally, there are other principles, that were beyond the scope of the present review, such as deliberate practice (Ericsson et al., 1993), and many aspects of healthy lifestyles (Walsh, 2011; Sigman et al., 2014) that also promote cognitive fitness. Integrating these approaches with good design could lead to a more comprehensive impact on WM function that might ultimately transfer to real-world conditions.

## Conflict of interest statement

The authors declare that the research was conducted in the absence of any commercial or financial relationships that could be construed as a potential conflict of interest.

## Footnotes

^1^https://itunes.apple.com/us/app/recall-the-game/id890271623?mt=8
